# SIRT7 ameliorates Th17/Treg imbalance by desuccinylation of STAT3 to improve immune thrombocytopenia

**DOI:** 10.1002/cti2.70048

**Published:** 2025-07-15

**Authors:** Jiao Ge, Xiaoyan Zhang, Fajuan Tang, Yan Liu

**Affiliations:** ^1^ Department of Emergency West China Second University Hospital, Sichuan University Chengdu China; ^2^ Key Laboratory of Birth Defects and Related Diseases of Women and Children Ministry of Education, Sichuan University Chengdu China

**Keywords:** immune thrombocytopenia, SIRT7, STAT3, succinylation, Th17, Treg

## Abstract

**Objectives:**

The imbalance of Th17/Treg cells represents a key pathogenic mechanism in immune thrombocytopenia (ITP); however, the underlying regulatory mechanisms remain poorly understood. Dysregulated succinylation has been implicated in disease onset and progression. Therefore, this study aimed to investigate the role of succinylation in modulating the Th17/Treg balance in ITP and to elucidate the associated molecular pathways.

**Methods:**

Whole blood samples were collected from ITP patients and mouse models. The frequencies of Treg and Th17 cells were quantified using flow cytometry. Treg‐ and Th17‐associated biomarkers were analysed via enzyme‐linked immunosorbent assay, quantitative real‐time polymerase chain reaction and immunoblotting. The regulatory relationship between SIRT7 and STAT3 succinylation was evaluated through co‐immunoprecipitation, immunofluorescence and immunoblotting assays.

**Results:**

Patients with ITP exhibited elevated Th17/Treg ratios, accompanied by increased global succinylation levels and reduced SIRT7 expression. Overexpression of SIRT7 restored the Th17/Treg imbalance *in vitro*. Mechanistically, SIRT7 overexpression suppressed STAT3 succinylation at K573, thereby inhibiting STAT3 activity and downstream signalling. Conversely, enforced STAT3 expression counteracted the effects of SIRT7 overexpression on Th17/Treg dynamics. *In vivo* experiments demonstrated that SIRT7 knockout exacerbated thrombocytopenia and further disrupted Th17/Treg homeostasis in murine models.

**Conclusion:**

SIRT7 mitigates ITP progression by maintaining Th17/Treg equilibrium through desuccinylation of STAT3. These findings highlight SIRT7 as a potential therapeutic target for ITP treatment, offering novel insights into the epigenetic regulation of immune dysregulation in autoimmune diseases.

## Graphical abstract

In this study, we investigated the effect of SIRT7 on the balance of Th17/Treg in immune thrombocytopenia (ITP) and to elucidate the associated molecular pathways. We found that SIRT7 improves the imbalance of Th17/Treg cells by inhibiting succinylation of STAT3, thereby ameliorating the progression of ITP, suggesting novel insights into the epigenetic regulation of immune dysregulation.

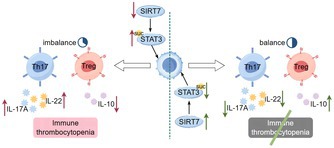

## Introduction

Immune thrombocytopenia (ITP) is an autoimmune disorder characterised by immune‐mediated platelet destruction, leading to markedly reduced platelet counts and an elevated risk of bleeding.^1^ ITP exhibits significant clinical heterogeneity, ranging from asymptomatic or mild mucosal bleeding to severe, life‐threatening haemorrhage.^2^ Although the aetiology of ITP is multifactorial, involving genetic predispositions and environmental triggers,^3^ aberrant immune responses play a central role in its pathogenesis.^4^ Among the diverse immune dysregulations observed in ITP, the imbalance between regulatory T cells (Tregs) and T helper 17 (Th17) cells has emerged as a critical contributor to disease progression.^5^ Tregs, defined by the expression of the transcription factor FOXP3, are essential for maintaining immune homeostasis through the production of anti‐inflammatory cytokines. In contrast, Th17 cells, characterised by the secretion of IL‐17, exhibit pro‐inflammatory and pro‐autoimmune functions.^6^, ^7^ Thus, the equilibrium between Tregs and Th17 cells is vital for immune homeostasis, and its disruption may precipitate autoimmune pathologies.

Post‐translational modifications (PTMs) are critical regulatory mechanisms that modulate protein function, localisation and stability.^8^ These modifications govern diverse cellular processes, including proliferation, apoptosis, angiogenesis, metastasis, inflammation and immune responses.^9^ Succinylation, a relatively recently identified PTM, has been shown to alter the structure and function of various proteins. This modification is dynamically regulated by succinyltransferases and desuccinylases.^10^ Sirtuin 7 (SIRT7), a member of the NAD^+^‐dependent deacetylase family that also functions as a desuccinylase, has been implicated in multiple pathological conditions, such as cancer, cardiac fibrosis, asthma, gastrointestinal disorders and inflammatory diseases.^11^ Recent studies have suggested a potential role for SIRT7 in autoimmune diseases.^12^ However, the specific contributions of succinylation—and particularly the desuccinylase activity of SIRT7—to the pathogenesis of ITP remain poorly characterised.

Signal Transducer and Activator of Transcription 3 (STAT3) is a transcription factor critical for maintaining tissue homeostasis and regulating inflammatory and immune responses. Genetic mutations, hyperactivation or hypoactivation of STAT3 are linked to immunodeficiency and autoimmune disorders.^13^ Importantly, STAT3 directly governs the differentiation and functional activity of regulatory Tregs and Th17 cells.^14^, ^15^ Recent studies have demonstrated that STAT3 is aberrantly activated in ITP, and pharmacological interventions can suppress its activation.^16^, ^17^ However, the potential role of succinylation in modifying STAT3 to regulate Th17/Treg cell dynamics in ITP remains unexplored.

This study aimed to investigate the role of SIRT7‐mediated desuccinylation in modulating the Th17/Treg balance in ITP, with a specific focus on the succinylation status of STAT3. Our findings demonstrate that SIRT7 exerts a critical regulatory function in ITP by desuccinylating STAT3, thereby restoring the Th17/Treg equilibrium. These results highlight SIRT7 as a potential therapeutic target for ITP, offering new insights into the molecular mechanisms underlying immune dysregulation in this disorder.

## Results

### Th17/Treg balance is disrupted in patients with ITP

**Figure 1:**
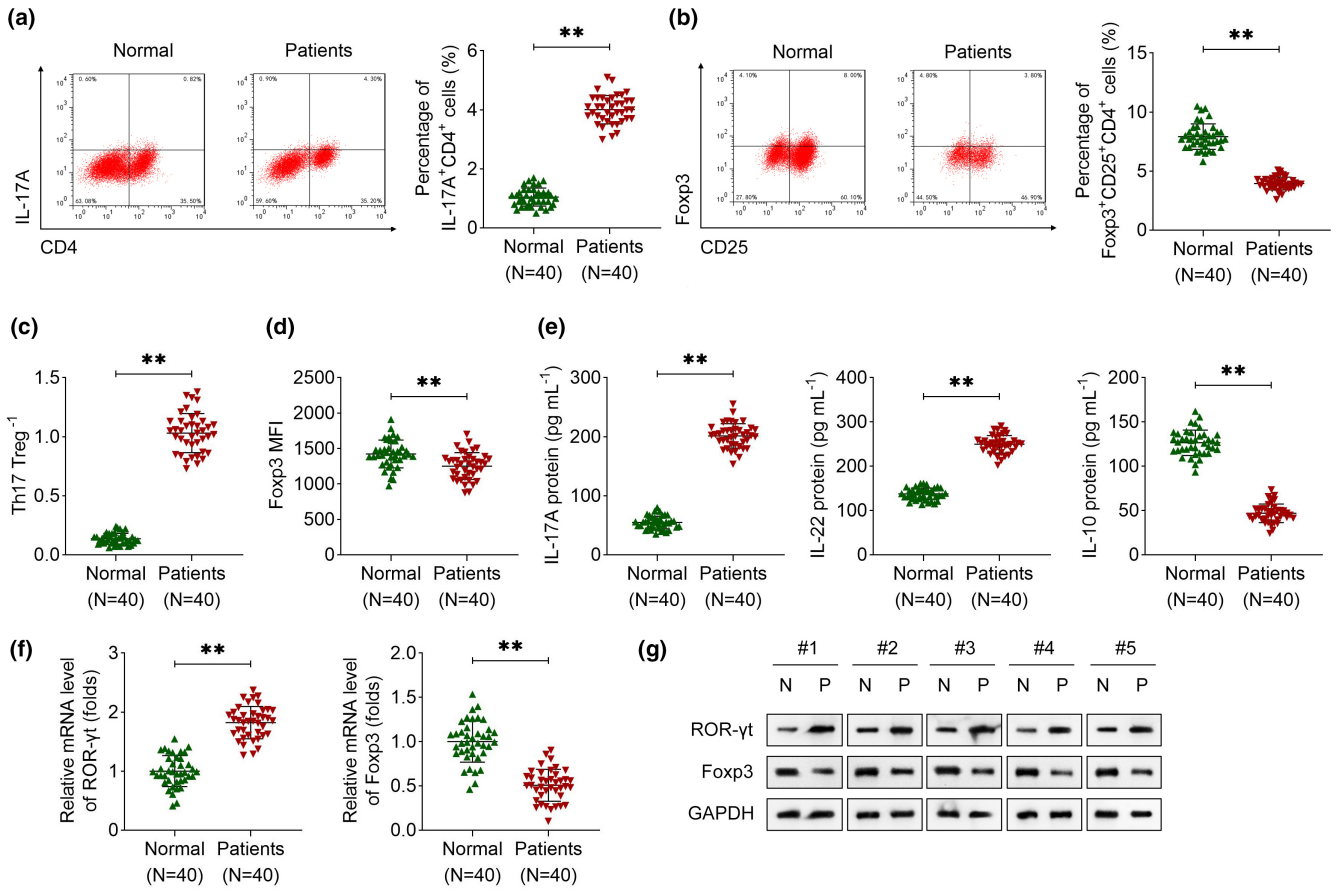
Th17/Treg balance is disrupted in patients with ITP. **(a)** Th17 (IL17A^+^CD4^+^) cells and **(b)** Treg (Foxp3^+^CD25^+^CD4^+^) cells in patients with ITP and normal participants were measured by flow cytometry using PBMCs. The percentage of Th17 or Treg cells was calculated according to the number of PBMCs. **(c)** Th17/Treg ratio was calculated. **(d)** Foxp3 MFI results were acquired from flow cytometry. **(e)** The levels of IL‐17A, IL‐22 and IL‐10 in the blood were measured by ELISA. **(f)** ROR‐γt and Foxp3 expression in the blood sample was detected using qRT‐PCR. **(g)** ROR‐γt and Foxp3 protein levels were examined using immunoblotting. Protein bands of five samples are shown. *n* = 40 per group in each experiment. Each sample in each experiment was technically replicated three times. **P < 0.01.

To investigate alterations in Treg and Th17 cells in ITP, we collected peripheral blood samples from patients with ITP and healthy controls. PBMCs were isolated from the blood sample, and flow cytometry was performed to measure the percentage of Treg (Foxp3^+^CD25^+^CD4^+^) and Th17 (IL‐17A^+^CD4^+^) cells. The results showed that the percentage of IL‐17A^+^CD4^+^ cells was increased, and the percentage of Foxp3^+^ CD25^+^ CD4^+^ cells was reduced in patients with ITP (Figure 1a and b). Thus, the Th17/Treg ratio was increased in ITP (Figure 1c). Foxp3 MFI was lower in the ITP group than that in the normal group (Figure 1d). Next, ELISA results demonstrated that the plasma concentrations of IL‐17A and IL‐22 were significantly elevated, whereas IL‐10 levels were markedly reduced in ITP patients compared with healthy controls (Figure 1e). To further validate these findings, qRT‐PCR and immunoblotting analyses were conducted to assess the expression levels of ROR‐γt (Th17 marker) and Foxp3 (Treg marker). Both mRNA and protein levels of ROR‐γt were upregulated in the blood of ITP patients, while Foxp3 expression was downregulated at both the transcriptional and translational levels (Figure 1f and g). The results demonstrate that Th17 cells were increased in patients with ITP, while Treg cells were reduced.

### SIRT7 expression is downregulated in patients with ITP

**Figure 2:**
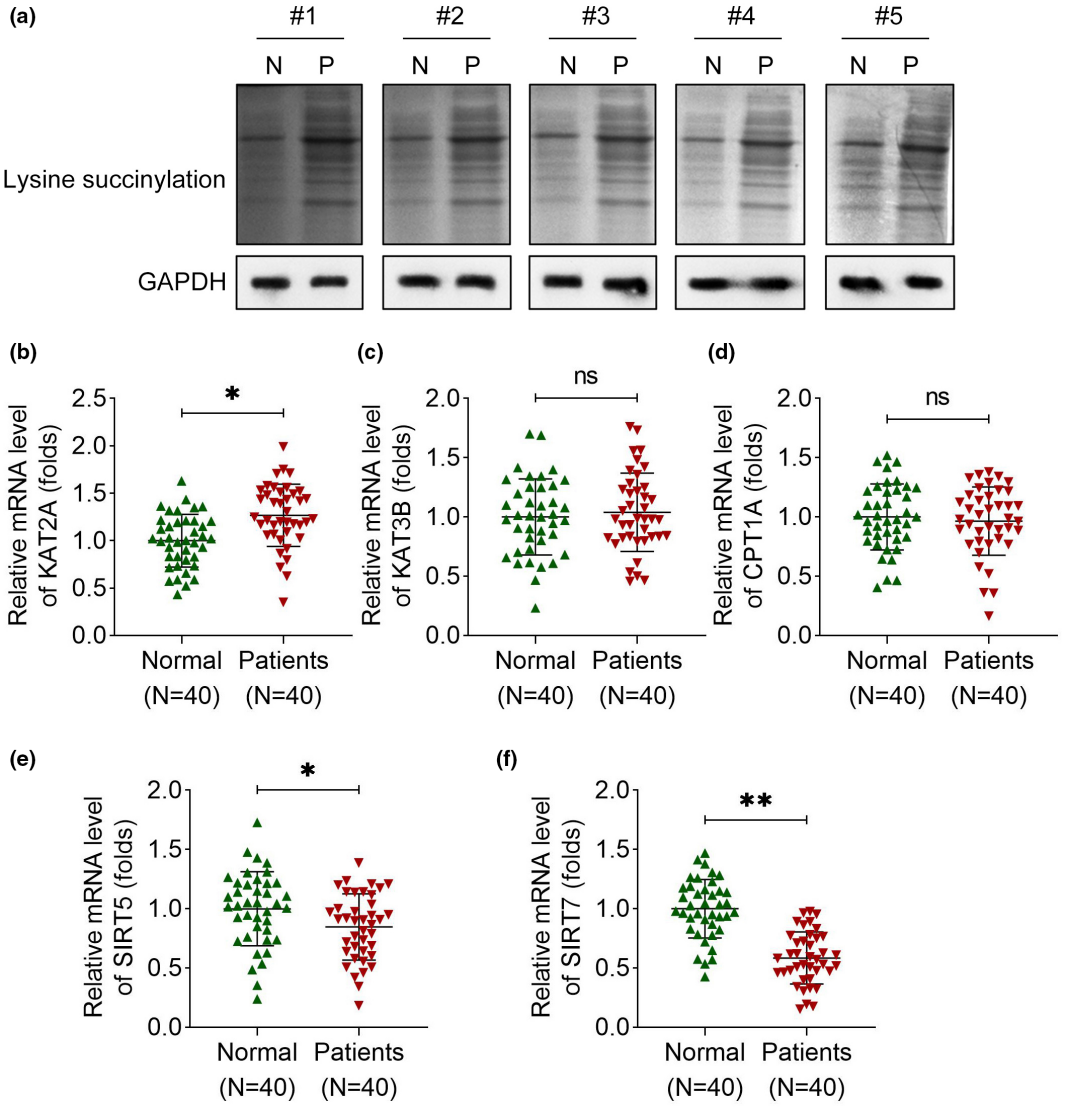
SIRT7 expression is downregulated in patients with ITP. **(a)** Total succinylation levels in the blood samples of all participants were measured using immunoblotting. Five bands were shown in this figure. **(b)** KAT2A, **(c)** KAT3B, **(d)** CPT1A, **(e)** SIRT5 and **(f)** SIRT7 expression in the blood of patients with ITP and normal individuals was detected using qRT‐PCR by normalising to internal control GAPDH expression. Data are presented as fold change relative to the mean expression level in healthy controls. *n* = 40 per group in each experiment. Each sample in each experiment was technically replicated three times. **P < 0.01; *P < 0.05; ns, no significance.

To determine whether succinylation contributes to ITP, we first quantified total lysine succinylation levels in peripheral blood samples from participants. Lysine succinylation was significantly elevated in the blood of ITP patients compared with healthy controls (Figure 2a). Next, we analysed the expression levels of key succinylation‐related enzymes. Our results showed that KAT2A was upregulated in ITP patients, whereas SIRT5 and SIRT7 exhibited reduced expression in the same cohort (Figure 2b, e and f). In contrast, KAT3B and CPT1A showed no significant differences between the two groups (Figure 2c and d). These findings indicate that ITP is associated with global succinylation enhancement, accompanied by increased KAT2A expression and decreased SIRT5/SIRT7 activity. Based on the most pronounced differences in expression levels, we selected SIRT7 for further investigation.

### Overexpression of SIRT7 improves the imbalance of Th17/Treg cells

**Figure 3:**
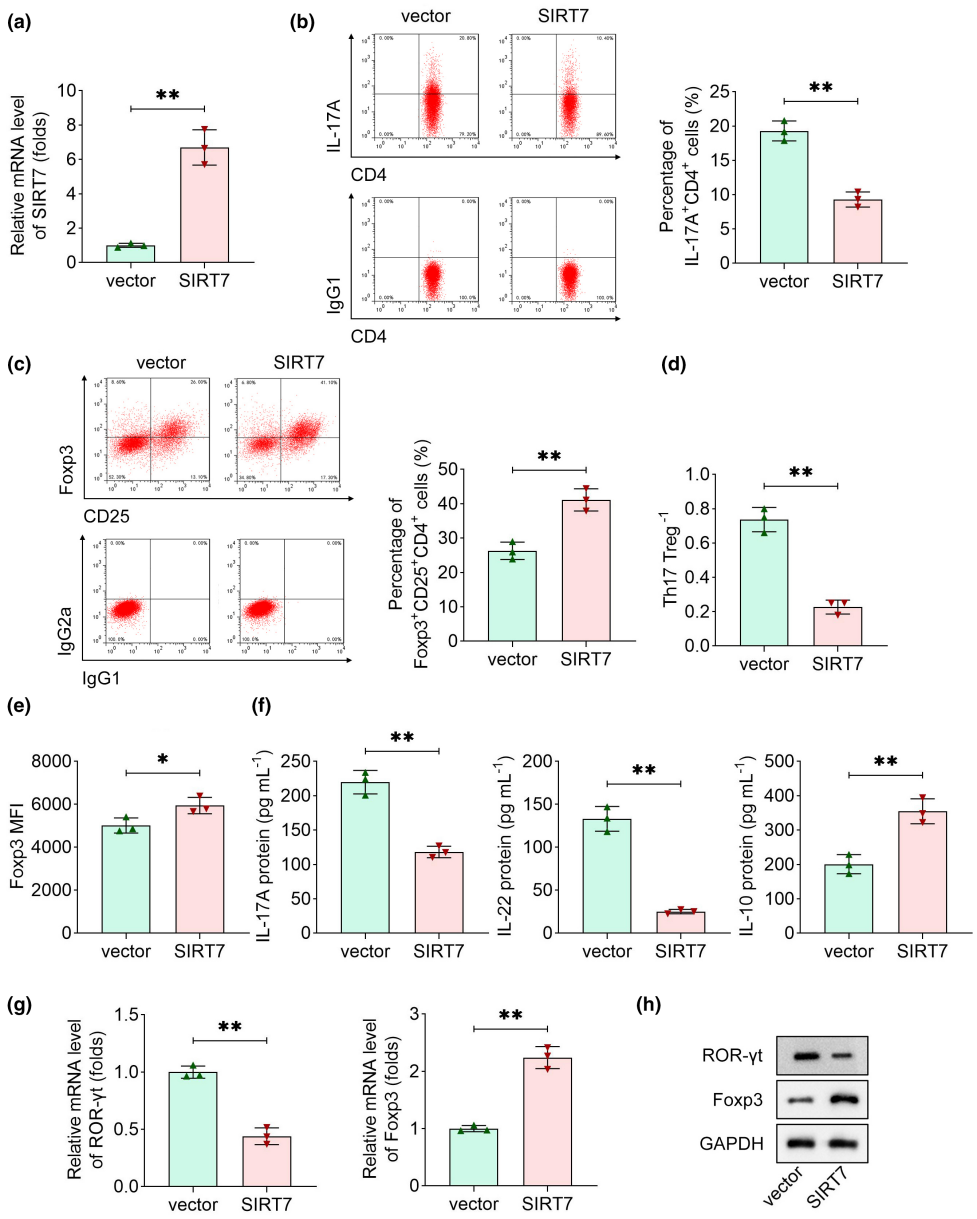
Overexpression of SIRT7 improves the imbalance of Trea/Th17 cells. **(a)** SIRT7 expression in CD4^+^ T cells was measured after transfection. **(b)** Th17 (IL17A^+^CD4^+^) and **(c)** Treg (Foxp3^+^CD25^+^CD4^+^) cells were measured using flow cytometry. The percentage of Th17 or Treg cells was calculated according to the number of CD4^+^ T cells. **(d)** Th17/Treg ratio was quantified. **(e)** Foxp3 MFI results were acquired from flow cytometry. **(f)** ELISA was performed to determine the levels of IL‐17A, IL‐22 and IL‐10. **(g)** qRT‐PCR and **(h)** immunoblotting were carried out to detect the expression of ROR‐γt and Foxp3. *n* = 3 per group in each experiment. Each sample in each experiment was technically replicated three times. **P < 0.05; **P < 0.01.

To investigate the effect of SIRT7 on Th17/Treg cells, we transfected empty vectors and SIRT7 overexpression plasmids into CD4^+^ T cells. As shown in Supplementary figure 1a, about 40% of cells were successfully transfected with plasmids. SIRT7 overexpression was confirmed in CD4^+^ T cells following plasmid transfection (Figure 3a). Functional analysis revealed that SIRT7 overexpression significantly reduced the percentage of Th17 cells and the Th17/Treg ratio, while concurrently increasing the proportion of Treg cells (Figure 3b–d). Foxp3 MFI was elevated by SIRT7 overexpression (Figure 3e). Cell culture supernatants were collected for cytokine quantification via ELISA. SIRT7 overexpression led to a marked decrease in IL‐17A and IL‐22 levels, accompanied by a significant increase in IL‐10 production (Figure 3f). Additionally, SIRT7 downregulated the expression of ROR‐γt and upregulated Foxp3 at both the mRNA and protein levels (Figure 3g and h). In summary, SIRT7 increased Treg cells and reduced Th17 cells.

### SIRT7 inhibits the succinylation of STAT3 at the K573 site

**Figure 4:**
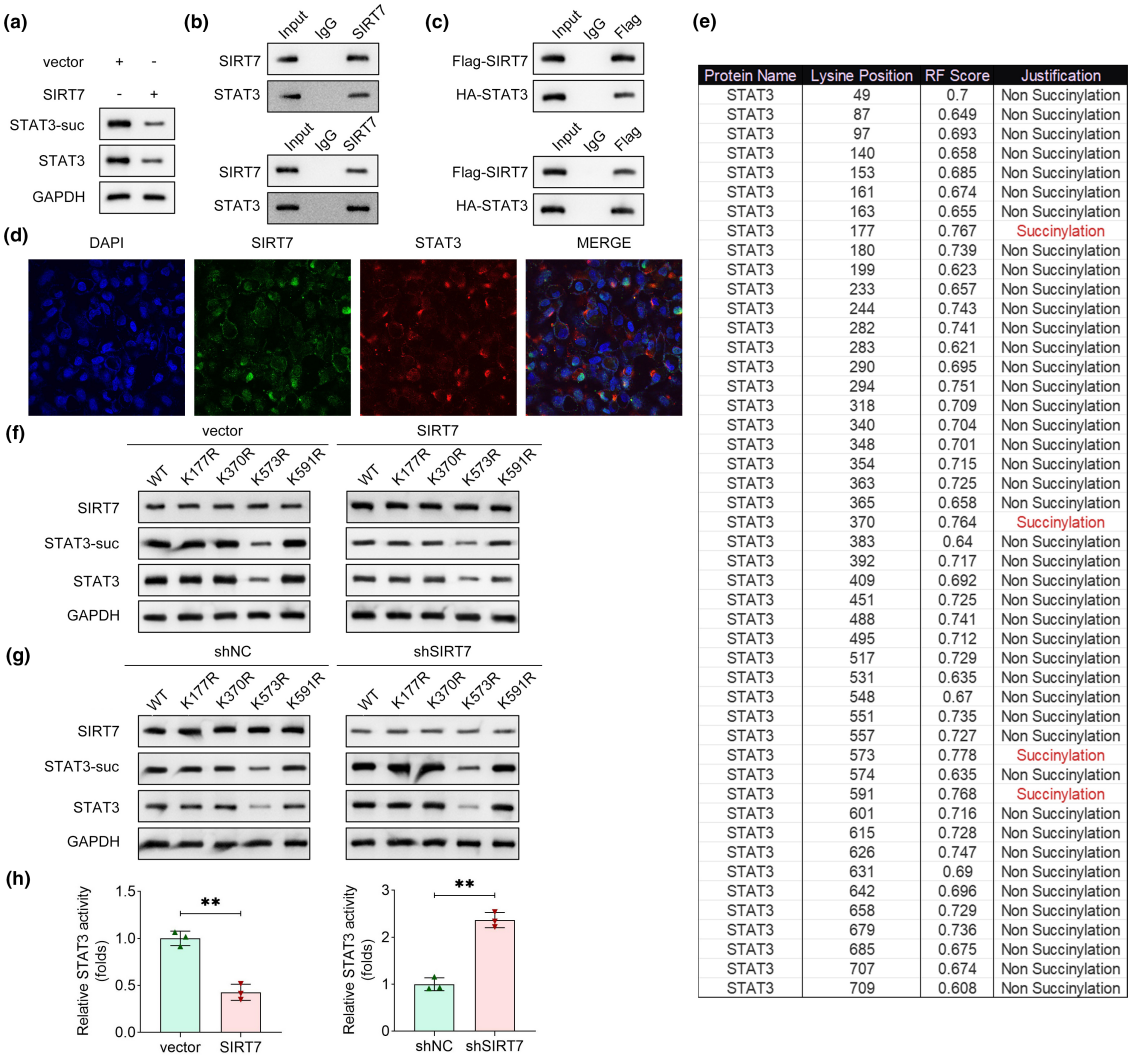
SIRT7 inhibits the succinylation of STAT3 at the K573 site. **(a)** The succinylation and protein levels of STAT3 were measured using immunoblotting after overexpressing SIRT7. **(b)** Endogenous or **(c)** exogenous co‐IP was performed to evaluate the interaction between SIRT7 and STAT3 proteins in HEK293T cells. **(d)** Co‐location of SIRT7 and STAT3 in HEK293T cells was viewed using immunofluorescence. **(e)** The possible succinylation sites in STAT3 were predicted using the GPSuc online tool. **(f, g)** After SIRT7 overexpression or knockdown, the succinylation sites in STAT3 were confirmed in cells that were mutated at these sites. **(h)** The activity of STAT3 was measured using a dual‐luciferase reporter assay after SIRT7 overexpression or knockdown. *n* = 3 per group in each experiment. Each sample in each experiment was technically replicated three times. **P < 0.01.

To evaluate SIRT7's role as a desuccinylase in regulating STAT3 succinylation, we first assessed the effects of SIRT7 overexpression on STAT3 modification. SIRT7 overexpression significantly reduced STAT3 succinylation levels and concurrently suppressed total STAT3 protein abundance (Figure 4a). Endogenous and exogenous co‐immunoprecipitation (co‐IP) experiments confirmed a direct interaction between SIRT7 and STAT3 (Figure 4b and c). Furthermore, immunofluorescence imaging revealed partial co‐localisation of SIRT7 and STAT3 in HEK293T cells (Figure 4d). These findings collectively demonstrate that SIRT7 physically interacts with STAT3 to desuccinylate it. To identify potential succinylation sites on STAT3, we conducted bioinformatics predictions, and the results are shown in Figure 4e. To validate these sites, we transfected WT or K573R mutant plasmids into CD4^+^ T cells. SIRT7 overexpression reduced both succinylation and total protein levels of STAT3, whereas SIRT7 knockdown induced the converse effects (Figure 4f and g). Notably, in the K573R group, STAT3 succinylation and protein levels were significantly lower than the WT group, confirming that K573 is a critical succinylation site. To further investigate the functional consequences of SIRT7‐mediated desuccinylation, we performed dual‐luciferase reporter assays using a STAT3 promoter‐luciferase reporter construct. SIRT7 overexpression significantly suppressed STAT3 transcriptional activity, while SIRT7 knockdown enhanced it (Figure 4h). Collectively, these data indicate that SIRT7 desuccinylates STAT3 at the K573 site, thereby reducing STAT3 expression by inhibiting its transcriptional activity.

### Overexpression of STAT3 reverses the impact on Th17/Treg cells modulated by SIRT7

**Figure 5:**
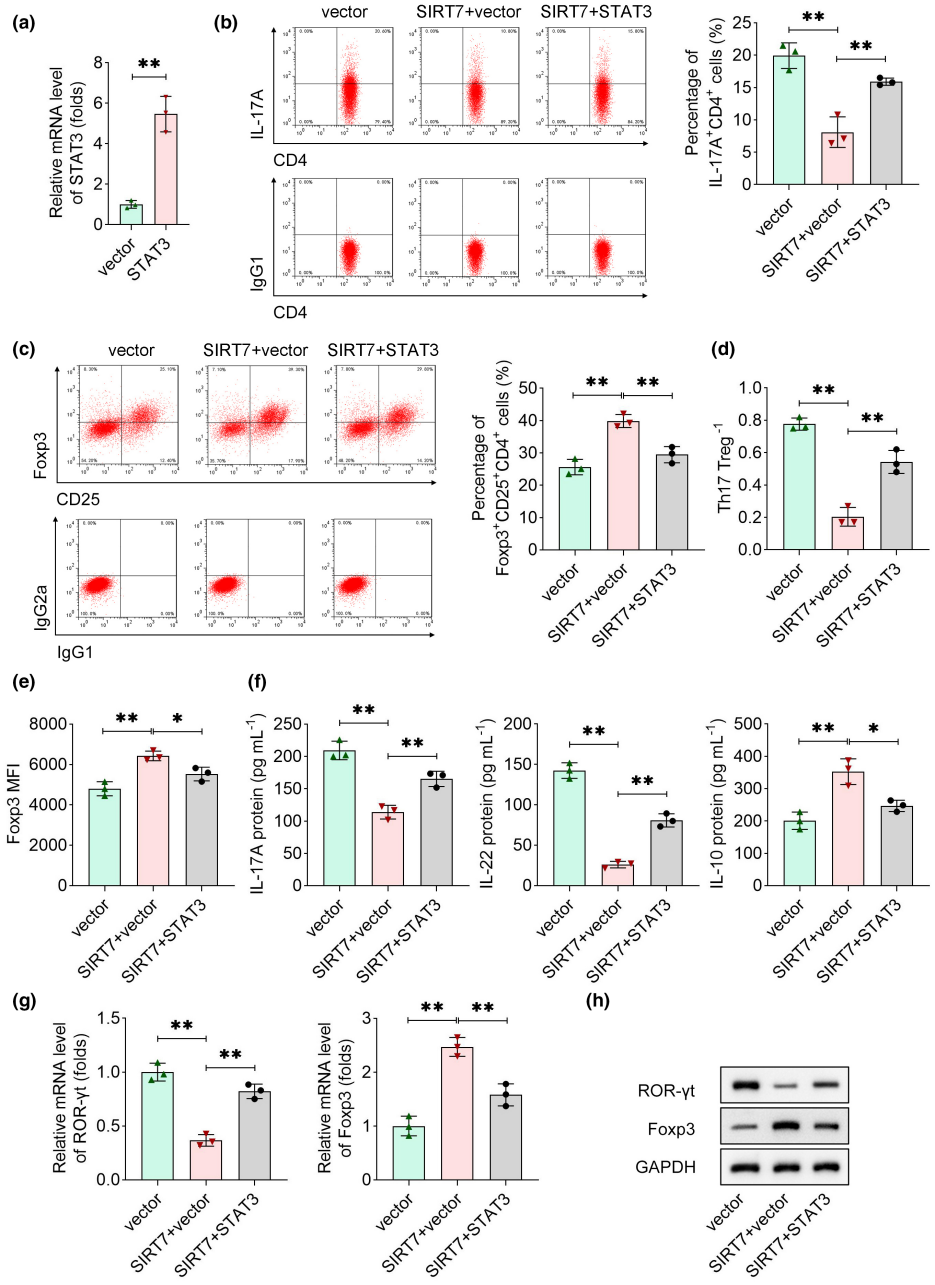
Overexpression of STAT3 reversed the impact on Th17/Treg cells modulated by SIRT7. **(a)** STAT3 expression was measured by qRT‐PCR after transfecting with its overexpression plasmids. **(b)** Th17 (IL17A^+^CD4^+^) and **(c)** Treg (Foxp3^+^CD25^+^CD4^+^) cells were measured using flow cytometry. The percentage of Th17 or Treg cells was calculated according to the number of CD4^+^ T cells. **(d)** Th17/Treg ratio was quantified. **(e)** Foxp3 MFI results were acquired from flow cytometry. **(f)** The levels of IL‐17A, IL‐22 and IL‐10 were detected using ELISA. **(g)** qRT‐PCR and **(h)** immunoblotting were carried out to detect the expression of ROR‐γt and Foxp3. *n* = 3 per group in each experiment. Each sample in each experiment was technically replicated three times. **P < 0.01; *P < 0.05.

The impact of STAT3 on Th17/Treg cells was evaluated using rescue experiments. The results of transfection efficiency showed that more than 30% of the cells were successfully transfected (Supplementary figure 1b). STAT3 overexpression was confirmed in CD4^+^ T cells transfected with the STAT3 overexpression plasmid (Figure 5a). Functional assays demonstrated that SIRT7 overexpression reduced Th17 cell populations and increased Treg cell proportions, an effect that was counteracted by STAT3 overexpression (Figure 5b and c). Consequently, the Th17/Treg ratio reduction induced by SIRT7 was reversed by STAT3 overexpression (Figure 5d). Overexpression of SIRT7 increased Foxp3 MFI, which was abrogated by STAT3 overexpression (Figure 5e). Cytokine quantification via ELISA further revealed that STAT3 overexpression counteracted the SIRT7‐mediated decreases in IL‐17A and IL‐22 and the increase in IL‐10 (Figure 5f). Moreover, SIRT7‐driven downregulation of ROR‐γt and upregulation of Foxp3 were partially reversed by STAT3 overexpression at both the mRNA and protein levels (Figure 5g and h). Collectively, these findings demonstrate that SIRT7 regulates Th17/Treg cell balance by suppressing STAT3 expression.

### Overexpression of STAT3 reduces Treg cells by inhibiting Foxp3 transcription

STAT3 is a transcription factor. Hence, we evaluated how STAT3 affected Treg cells by exploring the transcription of Foxp3. The results of chromatin immunoprecipitation (ChIP) showed that overexpression of STAT3 decreased its enrichment in the Foxp3 promoter (Supplementary figure 2a). Then, STAT3 reduced Foxp3 transcriptional activity (Supplementary figure 2b). Moreover, mRNA expression of Foxp3 was downregulated by STAT3 overexpression (Supplementary figure 2c).

### Knockout of SIRT7 exacerbates thrombocytopenia by promoting Th17/Treg imbalance

**Figure 6:**
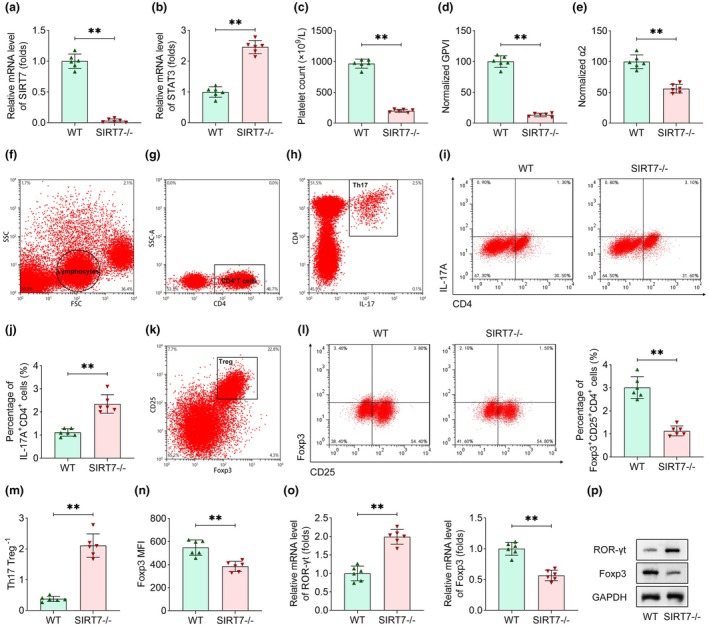
Knockout of SIRT7 exacerbates thrombocytopenia by promoting Th17/Treg imbalance. **(a)** SIRT7 and **(b)** STAT3 expression levels were detected in the blood of WT and SIRT7^−/−^ mice. **(c)** The number of platelets was counted. The expression of **(d)** GPVI and **(e)** α2 was measured using flow cytometry. **(f)** Gating strategy for lymphocytes. **(g)** Gating strategy for CD4^+^ T cells. **(h)** Gating strategy for Th17 cells. **(i, j)** Th17 (IL17A^+^CD4^+^) cells were measured by flow cytometry. The percentage of Th17 cells was calculated according to the number of PBMCs. **(k)** Gating strategy for Treg cells. **(l)** Treg (Foxp3^+^CD25^+^CD4^+^) cells were measured by flow cytometry. The percentage of Treg cells was calculated according to the number of PBMCs. **(m)** Th17/Treg ratio was calculated. **(n)** Foxp3 MFI results were acquired from flow cytometry. **(o)** ROR‐γt and Foxp3 at mRNA levels and **(p)** their protein levels were analysed using qRT‐PCR and immunoblotting, respectively. *n* = 6 per group in each experiment. Each sample in each experiment was technically replicated three times. **P < 0.01.

The effects of SIRT7 on ITP progression and Th17/Treg cells *in vivo* were analysed. WT and SIRT7^−/−^ mice were used to establish the ITP model. SIRT7 expression was detected exclusively in the whole blood of WT mice but was absent in SIRT7^−/−^ mice (Figure 6a). Notably, STAT3 expression was significantly elevated in SIRT7^−/−^ mice compared with WT controls (Figure 6b). SIRT7 deficiency led to a marked reduction in platelet counts and decreased expression of platelet surface markers, including GPVI and integrin α2 (Figure 6c–e). Then, Treg and Th17 cells were evaluated using flow cytometry. The gating strategy results are shown in Figure 6f–h and k. SIRT7 knockout resulted in increased Th17 cell proportions and decreased Treg cell frequencies, accompanied by a corresponding rise in the Th17/Treg ratio (Figure 6i, j, l and m). Foxp3 MFI was decreased in the SIRT7^−/−^ group, compared with the WT group (Figure 6n). Molecular analyses further demonstrated that SIRT7 deficiency upregulated ROR‐γt expression and downregulated Foxp3 at both the mRNA and protein levels (Figure 6o and p). Collectively, these findings indicate that SIRT7 knockout exacerbates ITP progression and disrupts Th17/Treg balance through STAT3‐dependent mechanisms.

## Discussion

Our study offers new mechanistic insights into the role of SIRT7‐mediated desuccinylation in the pathogenesis of ITP, highlighting the critical regulatory function of PTMs in immune homeostasis. We reveal that SIRT7 ameliorates ITP progression by desuccinylating STAT3, a process that subsequently restores the equilibrium between Treg and Th17 cell populations. These findings significantly advance our understanding of molecular pathways governing immune regulation and the underlying mechanisms of autoimmune disease pathogenesis.

Although multiple T‐cell subsets contribute to immune regulation in the host, Treg and Th17 cells remain the most critical players in immune homeostasis. The delicate balance between Treg and Th17 cells is essential for maintaining immune homeostasis, as its disruption is linked to various diseases, including autoimmune disorders. An elevated Th17/Treg ratio serves as a potential diagnostic biomarker for ITP.^18^ Furthermore, recent studies have focussed on strategies to address the Th17/Treg imbalance in ITP.^19^, ^20^, ^21^ Therefore, targeting the Th17/Treg axis may represent a promising therapeutic strategy for ITP.

SIRT7 has been implicated in the pathogenesis and therapeutic modulation of autoimmune diseases.^12^, ^22^ For instance, SIRT7 knockdown enhances melanin synthesis, which may hold therapeutic potential for vitiligo.^23^ Subtle immune dysregulation in SIRT7 knockout models of experimental autoimmune encephalomyelitis is characterised by impaired survival of newborn neurons.^24^ However, the role of SIRT7 in ITP remains unexplored. Our study is the first to investigate the functional contribution of SIRT7 to ITP progression. We observed elevated lysine succinylation levels in ITP patients, accompanied by reduced SIRT7 expression. *In vitro* and *in vivo* experiments revealed that SIRT7 overexpression increased Treg cell populations while decreasing Th17 cells, indicating a restoration of Th17/Treg balance. Furthermore, SIRT7 deficiency exacerbated thrombocytopenia and Th17/Treg imbalance *in vivo*. These findings establish SIRT7 as a critical regulator of immune homeostasis and suggest that targeting SIRT7‐mediated desuccinylation may represent a novel therapeutic strategy for ITP with disrupted Th17/Treg dynamics. In addition, we also found that KAT2A expression was elevated and SIRT5 expression was decreased in patients with ITP, suggesting that they play a crucial role in ITP by regulating succinylation. KAT2A has been revealed to regulate Treg functions. Specifically, its deficiency leads to a decrease in the stability and quantity of Treg cells, contributing to autoimmunity.^25^ SIRT5 has been reported to inhibit T‐cell activation in colorectal cancer, particularly regulating the differentiation of Treg and Th1 cells.^26^ These studies indicate that KAT2A and SIRT5 are closely related to Treg functions. However, their roles in ITP and the regulatory mechanism of Th17/Treg remain unknown and deserve further exploration in our future studies.

In the present study, the desuccinylation of STAT3 mediated by SIRT7 emerges as a critical regulator of Treg and Th17 cell dynamics. Specifically, SIRT7 inhibits STAT3 succinylation, suppresses STAT3 activity and consequently reduces STAT3 expression. Elevated STAT3 expression has been consistently observed in patients with ITP,^27^ and its dysregulation has been implicated in ITP pathogenesis through multiple mechanisms. For instance, Li *et al*.^28^ demonstrated that long noncoding RNA GAS5 inhibits Th17 differentiation without affecting Treg differentiation by promoting STAT3 ubiquitination. Similarly, Zhang *et al*.^29^ reported that daucosterol enhances megakaryocyte differentiation and platelet recovery by inactivating the JAK2/STAT3 signalling pathway. Crucially, STAT3 exerts divergent effects on Treg and Th17 cells across disease contexts. In periodontitis, STAT3 signalling activation skews the Th17/Treg balance towards Th17 differentiation,^15^ whereas STAT3 inactivation in gastric cancer promotes Th17 differentiation while suppressing Treg differentiation.^30^ Given its dual role, tight regulation of STAT3 activity is essential for maintaining the balance between Tregs and Th17 cells. Herein, we found that overexpression of STAT3 reversed the improvement of Th17/Treg balance mediated by SIRT7, suggesting that SIRT7 downregulates STAT3 expression to prevent Th17/Treg imbalance. Our research further demonstrates that STAT3 increases the number of Th17 cells, which is consistent with previous studies indicating that STAT3 activates Th17 cell differentiation.^31^, ^32^ The reducing effect of STAT3 on the percentage of Treg cells may depend on its transcriptional inhibitory effect on Foxp3, which is consistent with previous research results that STAT3 may inhibit Foxp3 expression by direct binding.^33^ In addition, SIRT5 was also a desuccinylase that is downregulated in patients with ITP. Although the role of SIRT5 was not specifically investigated in this study, we hypothesise that it may function similar to SIRT7 in ITP and exhibit potential functional redundancy in regulating succinylation. Such functional redundancy might enable compensatory regulation of succinylation levels between them, thereby influencing the pathophysiology of ITP. In future studies, we will examine the functional redundancy between SIRT5 and SIRT7 in modulating STAT3 succinylation and immune responses, which will facilitate a comprehensive understanding of their mechanistic roles in ITP.

Although our study suggests that SIRT7/STAT3 participates in ITP by regulating Th17/Treg balance, we were unable to definitively determine whether this axis is dysregulated in ITP compared with its normal regulatory role in healthy individuals or whether it is specifically activated under ITP pathological conditions. Furthermore, current findings indicate that SIRT7 modulates STAT3 succinylation, thereby influencing Th17/Treg balance, leading us to hypothesise that SIRT7 may act as a driver in ITP pathogenesis. However, STAT3 may also transcriptionally suppress SIRT7 expression, potentially forming a feedback loop that exacerbates Th17/Treg imbalance. These unresolved issues need further investigation.

Several limitations remain unanswered. We used Foxp3^+^CD25^+^CD4^+^ and IL‐17A^+^CD4^+^cells to evaluate the balance of Treg and Th17 cells. Although this method has been widely adopted in many studies, relying solely on IL‐17A and Foxp3 as markers of Th17 and Treg cells may not fully reflect the true phenotypes and functions of these cells. The precise regulatory mechanisms governing SIRT7 activity in the context of ITP and its specificity for STAT3 desuccinylation remain to be fully elucidated. Additionally, the potential roles of other PTMs in modulating STAT3 activity and Th17/Treg cell balance warrant further comprehensive investigation.

In conclusion, our study elucidates the intricate relationship between lysine succinylation and immune regulation. We emphasise that SIRT7 ameliorates the imbalance of Th17/Treg cells by suppressing the succinylation of STAT3, thereby alleviating ITP. These findings provide a foundation for therapeutic interventions in ITP based on correcting Th17/Treg imbalance.

## Methods

### Participants and sample collection

Patients with ITP (*n* = 40) and healthy controls (*n* = 40) were enrolled in this study. All participants signed written informed consent before the study. Patients with ITP were diagnosed according to international recommendations.^34^, ^35^ Fasting venous blood (5 mL) was collected from all participants and stored in tubes containing heparin anticoagulant for subsequent analysis. This study was approved by the Ethics Committee of West China Second University Hospital and performed according to the principles of the Declaration of Helsinki.

### CD4^+^ T‐cell isolation and culture

Anticoagulated blood was diluted with Hanks' Balanced Salt Solution (HBSS). Peripheral blood mononuclear cells (PBMCs) were isolated from blood samples using Ficoll‐Paque™ density gradient centrifugation at 800 × *g* for 30 min. PBMCs were subsequently collected, washed with phosphate‐buffered saline (PBS), and resuspended for further processing. CD4^+^ T cells were purified from PBMCs using a human CD4^+^ T‐cell isolation kit (Miltenyi Biotec, Cologne, Germany) according to the manufacturer's instructions. Isolated CD4^+^ T cells were seeded into 24‐well plates pre‐coated with 5 μg mL^−1^ anti‐CD3 antibody and 1 μg mL^−1^ soluble anti‐CD28 antibody. Cells were cultured in Roswell Park Memorial Institute (RPMI)‐1640 medium (Gibco, Grand Island, NY, USA) supplemented with 10% fetal bovine serum (Gibco) and 1% penicillin–streptomycin (Gibco) at 37°C in a 5% CO_2_ incubator.

### Determination of Treg and Th17 cells

To detect the percentage of Th17 (IL‐17A^+^CD4^+^ cells) cells, PBMCs were stimulated by 1 μL of Phorbol 12‐Myristate 13‐Acetate (PMA)/Ionomycin mixture (Multi Science, Hangzhou, China) and 1 μL of Brefeldin A (BFA)/Monensin (Multi Science) at 37°C for 5 h. Then, PBMCs or CD4^+^ T cells were incubated with FITC‐conjugated anti‐CD4 monoclonal antibody in the dark for 30 min. After fixing and permeabilising using the BD Cytofix/Cytoperm™ Fixation/Permeabilisation solution kit (BD, Franklin Lakes, NJ, USA), the cells were subsequently incubated with APC‐conjugated anti‐IL17A monoclonal antibody in the dark for 30 min. To detect the percentage of Treg (Foxp3^+^CD25^+^CD4^+^) cells, PBMCs or CD4^+^ T cells were incubated with FITC‐conjugated anti‐CD4 monoclonal antibody and PE‐conjugated anti‐CD25 monoclonal antibody in the dark for 30 min, followed by incubation with APC‐conjugated Foxp3 monoclonal antibody after fixation and permeabilisation. All antibodies were purchased from eBioscience (San Diego, CA, USA). The detailed information of antibodies was as follows: anti‐CD4 (human Cat. No. 11‐0049‐42; mouse Cat. No. 11‐0042‐82), anti‐IL17A (human Cat. No. 17‐7179‐42, isotype control APC‐conjugated anti‐mouse IgG1 Cat. No. 17‐4714‐82; mouse Cat. No. 17‐7177‐81, isotype control APC‐conjugated anti‐rat IgG2a Cat. No. 17‐4321‐81), anti‐CD25 (human Cat. No. 12‐0259‐80, isotype control PE‐conjugated anti‐mouse IgG1 Cat. No. 12‐4714‐82; mouse Cat. No. 12‐0251‐82, isotype control PE‐conjugated anti‐rat IgG1 Cat. No. 12‐4301‐82) and anti‐Foxp3 (human Cat. No. 17‐4776‐42, isotype control APC‐conjugated anti‐rat IgG2a Cat. No. 17‐4321‐81; mouse Cat. No. 17‐5773‐82, isotype control APC‐conjugated anti‐rat IgG2a Cat. No. 17‐4321‐81). The samples were analysed using a flow cytometer. The percentage of Th17 or Treg cells = the number of IL‐17^+^ cells/the number of PBMCs/CD4^+^ T cells × 100%. The ratio of Th17/Treg cells = the percentage of Th17 cells/the percentage of Treg cells.

The gating strategy was as follows: Cells were gated by FSC/SSC to exclude dead cells and debris and identify lymphocytes. CD4^+^ cells were gated from the lymphocytes. IL‐17A^+^ and IL‐17A^−^ cells were distinguished according to the background signals of the isotype control. In addition, the CD4^+^ cell population was then gated for the CD25^+^ cells, and Foxp3^+^ cells were identified according to the background signals of the unstained control and the isotype control.

To detect Foxp3 mean fluorescence intensities (MFI), cells were incubated with APC‐conjugated Foxp3 monoclonal antibody and its corresponding isotype control. Each sample was analysed with a minimum of 10 000 cells using a flow cytometer. The corrected MFI = MFI (each sample) − MFI (isotype control).

### Enzyme‐linked immunosorbent assay (ELISA)

The levels of IL‐17A, IL‐22 and IL‐10 in the blood samples of participants and the cell culture supernatant were measured using their corresponding commercial kits (Elabscience, Wuhan, China).

### Quantitative real‐time polymerase chain reaction (qRT‐PCR)

Total RNA was extracted from whole blood using the Whole Blood Total RNA Kit (Sigmen, Hangzhou, China) and from cultured cells using TRIzol reagent (Carlsbad, CA, USA). RNA concentration was determined by measuring absorbance at 260/280 nm, and RNA integrity was assessed via 1% agarose gel electrophoresis. One microgram of total RNA was used for cDNA synthesis of the first strand using the PrimeScript RT Reagent Kit (Takara, Tokyo, Japan). Quantitative PCR was then carried out using Power SYBR™ Green PCR Master Mix (Applied Biosystems, Foster City, CA, USA). The relative expression levels of target mRNAs were calculated using the 2^−ΔΔCT^ method. GAPDH served as the internal control. The primer sequences are provided in Supplementary table 1.

### Immunoblotting

CD4^+^ T cells were lysed using radioimmunoprecipitation assay lysis buffer, and the supernatant was collected after centrifugation. Total protein was extracted from blood samples using the Whole Blood Protein Extraction Kit (BestBio, Shanghai, China). Protein concentration was quantified using the Pierce™ BCA Protein Assay Kit (Thermo Fisher Scientific, Waltham, MA, USA). Equal amounts of protein were resolved via 10% sodium dodecyl sulfate‐polyacrylamide gel electrophoresis, transferred onto polyvinylidene fluoride membranes (Millipore, Billerica, MA, USA), and blocked with 5% skim milk. Membranes were incubated overnight at 4°C with primary antibodies against ROR‐γt (ab113434, Abcam, Cambridge, MA, USA), Foxp3 (ab215206, Abcam), GAPDH (ab9485, Abcam), succinyllysine (PTM‐401, PTMBio, Hangzhou, China), SIRT7 (ab259968, Abcam) and STAT3 (ab32500, Abcam). After washing, membranes were incubated with secondary antibodies (donkey anti‐goat IgG ab6885, goat anti‐rabbit IgG ab6721, Abcam) for 2 h. Protein bands were visualised using the Novex™ ECL Chemiluminescence Substrate Kit (Invitrogen).

### Cell transfection

The full length of SIRT7 and STAT3 was cloned into pEGFP‐N1 vectors to construct the SIRT7 overexpression plasmid and the STAT3 overexpression plasmid. The empty pEGFP‐N1 vector served as the negative control. These plasmids were transfected into CD4^+^ T cells in six‐well plates using Lipofectamine 2000 (Invitrogen) in line with the manufacturer's instructions.

After 24‐h transfection, the transfection efficiency was measured using flow cytometry. Briefly, the cells were washed and resuspended using PBS, and cell concentration was adjusted to 1 × 10^6^ cells mL^−1^. The cells were incubated with 10 μL of 50 μg mL^−1^ propidium iodide (PI) in the dark for 5 min. Each sample was analysed with a minimum of 10 000 cells using a flow cytometer. The gating strategy was as follows: Cells were gated by FSC/SSC to exclude dead cells and debris and identify lymphocytes. Single cells were gated based on FSC‐A and FSC‐H. Alive cells were gated based on the negative results of PI staining. GFP‐positive cells were selected, and their corresponding percentage was considered as the transfection efficiency.

### Co‐IP

The interaction between SIRT7 and STAT3 was assessed using the Co‐IP Kit (BersinBio, Guangzhou, China). HEK293T cells were lysed in cell lysis buffer supplemented with protease inhibitors. The supernatant was obtained after centrifugation and incubated with primary antibodies [anti‐SIRT7 (ab259968, Abcam), anti‐STAT3 (ab119352, Abcam), anti‐Flag (ab125243, Abcam), anti‐HA (ab1424, Abcam), or isotype control IgG (ab37355, Abcam)] overnight at 4°C. Subsequently, the antibody‐bound complexes were incubated with Protein A/G magnetic beads (BersinBio) for 2 h at 4°C. After extensive washing, the bound proteins were eluted from the beads. The eluted protein samples were analysed by immunoblotting as described above.

### Immunofluorescence

HEK293T cells were fixed in 4% paraformaldehyde and blocked in 5% bovine serum albumin (BSA) for 30 min. The cells were incubated with primary antibodies against SIRT7 (ab259968, Abcam) and STAT3 (ab119352, Abcam) at 4°C for one night and then incubated with fluorescence‐conjugated secondary antibodies (Alexa Fluor 488‐labelled goat anti‐rabbit: ab150077, Abcam; Alexa Fluor 647‐labelled goat anti‐mouse: ab150115, Abcam) at 37°C for 1 h. The nucleus was stained with DAPI, and images were taken using a laser scanning confocal microscope.

### Succinylation of STAT3 analysis

Following transfection, CD4^+^ T cells were lysed using IP lysis buffer and centrifuged to collect the supernatant. The lysate was incubated with anti‐STAT3 antibody and Protein A/G magnetic beads overnight at 4°C. After elution of the bound complexes from the beads, immunoblotting was performed to detect STAT3 succinylation levels using a lysine succinylation‐specific primary antibody.

Potential succinylation sites on STAT3 were predicted using the GPSuc database (http://kurata14.bio.kyutech.ac.jp/GPSuc/index.php). To validate these modification sites, wild‐type (WT) STAT3 plasmids, as well as plasmids carrying lysine‐to‐arginine mutations (K177R, K370R, K573R and K591R), were transfected into CD4^+^ T cells. Subsequently, STAT3 succinylation levels were analysed using the same immunoprecipitation and immunoblotting protocol described above.

### Determination of STAT3 and Foxp3 activity

The promoter region of STAT3 or Foxp3 was cloned into the pGL4Luc‐Rluc vector (Addgene, Watertown, MA, USA) to construct the luciferase reporter plasmids. To detect STAT3 activity, the STAT3 reporter plasmids were co‐transfected with vector or SIRT7 overexpression plasmid using Lipofectamine 2000. Additionally, the STAT3 reporter plasmids were co‐transfected with short hairpin RNA (shRNA) targeting SIRT7 (shSIRT7) and shRNA‐negative control (shNC) using the same reagent. To measure Foxp3 activity, the Foxp3 reporter plasmids were co‐transfected with vector or STAT3 overexpression plasmid. The firefly and *Renilla* luciferase activities were measured using a double‐luciferase reporter assay kit (Gene Create, Wuhan, China).

### ChIP

This experiment was performed using the Pierce magnetic ChIP kit (Thermo Fisher Scientific). Briefly, cells were incubated with 1% formaldehyde for 10 min and incubated with glycine solution for 5 min. Then, cells were lysed using a membrane extraction buffer, and cell nuclei were collected for ultrasonication. After centrifuging at 9000 × *g* for 5 min, the supernatant was collected. The supernatant (90 μL) was mixed with 410 μL IP dilution buffer and incubated with 10 μg anti‐IgG or anti‐STAT3 at 4°C for 2 h, followed by further incubation with protein A/G magnetic beads at 4°C for 2 h. The beads were washed with washing buffer and eluted using elution buffer. Decrosslinking was performed using 6 μL of 5 M NaCl containing 2 μL of 20 mg mL^−1^ protease K at 65°C for 90 min. DNA was purified and served as a template in qPCR. Foxp3 expression was detected using qPCR.

### Animal study

WT C57BL/6J mice (8–12 weeks old, male) and age‐ and sex‐matched SIRT7 knockout (SIRT7^−/−^) C57BL/6J mice were purchased from Cyagen (Suzhou, China). Mice were housed in a specific pathogen‐free facility with unrestricted access to food and water. All animal experiments were conducted in accordance with the ARRIVE guidelines. To induce ITP,^36^ mice in each group (*n* = 6) were intraperitoneally injected with 0.1 mg kg^−1^ anti‐CD41 monoclonal antibody (clone: MWReg30, catalogue No. MA5‐44030, Invitrogen). Twenty‐four hours after antibody administration, blood samples were collected via the tail vein. Platelet counts were determined using an automated haematology analyser.

### Detection of GPVI and α2 expression

Anticoagulated whole blood was collected from all mice and incubated with FITC‐conjugated anti‐mouse GPVI or α2 antibodies. Flow cytometry was performed to quantify the expression levels of these surface markers.

### Statistical analysis

The GraphPad Prism 8 software was used for data analysis, and the results were expressed as mean ± standard deviation. Differences between the two groups were analysed using the unpaired Student's *t*‐test, and those among multiple groups were analysed using one‐way analysis of variance (ANOVA). *P* < 0.05 was considered statistically significant.

## Ethics statement

The study was approved by the Ethics Committee of West China Second University Hospital. Written informed consent was obtained from all patients. All experiments were performed in accordance with relevant guidelines and regulations.

## Funding

The authors declare that no funds, grants or other support were received during the preparation of this manuscript.

## Author contributions

**Jiao Ge:** Data curation; formal analysis; Methodology; Investigation; writing – original draft. **Xiaoyan Zhang:** Formal analysis; Methodology; Resources; Visualization; writing – review and editing. **Fajuan Tang**: Data curation; Visualization; Investigation; Writing‐ Reviewing and Editing. **Yan Liu:** Conceptualization; writing – review and editing.

## Conflict of interest

The authors declare no conflict of interest.

## Supporting information


Supplementary figure 1
Supplementary figure 2
Supplementary table 1

## Data Availability

The datasets used and/or analysed during this study are available from the corresponding author upon reasonable request.
