# Supplementary material for: SIRT7 ameliorates Th17/Treg imbalance by desuccinylation of STAT3 to improve immune thrombocytopenia
Source: Clin Transl Immunology. 2025 Jul 15;14(7):e70048. doi: 10.1002/cti2.70048 (PMC12263510; doi:10.1002/cti2.70048)
Supplement: Supplementary file 1 — Supplementary figure 1 Supplementary figure 2 Supplementary table 1 [file CTI2-14-e70048-s001.docx]

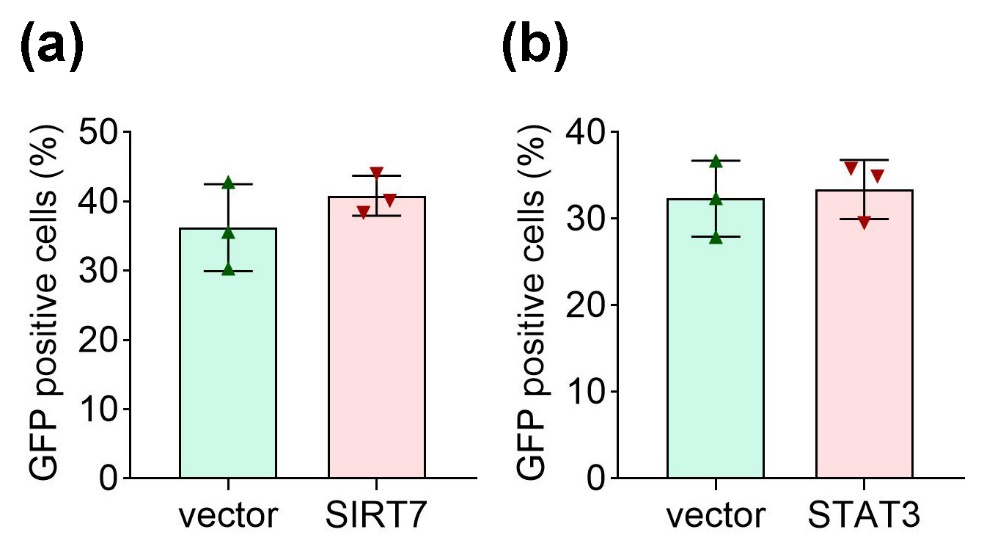


**Supplementary figure 1.** Transfection efficiency of overexpression plasmids. **(a)** SIRT7 overexpression plasmids, **(b)** STAT3 overexpression plasmids, and their negative control were transfected into CD4^+^ T cells, and transfection efficiency was detected using flow cytometry, calculated as the percentage of GFP positive cells. n = 3 per group in each experiment. Each sample in each experiment was technically replicated three times.


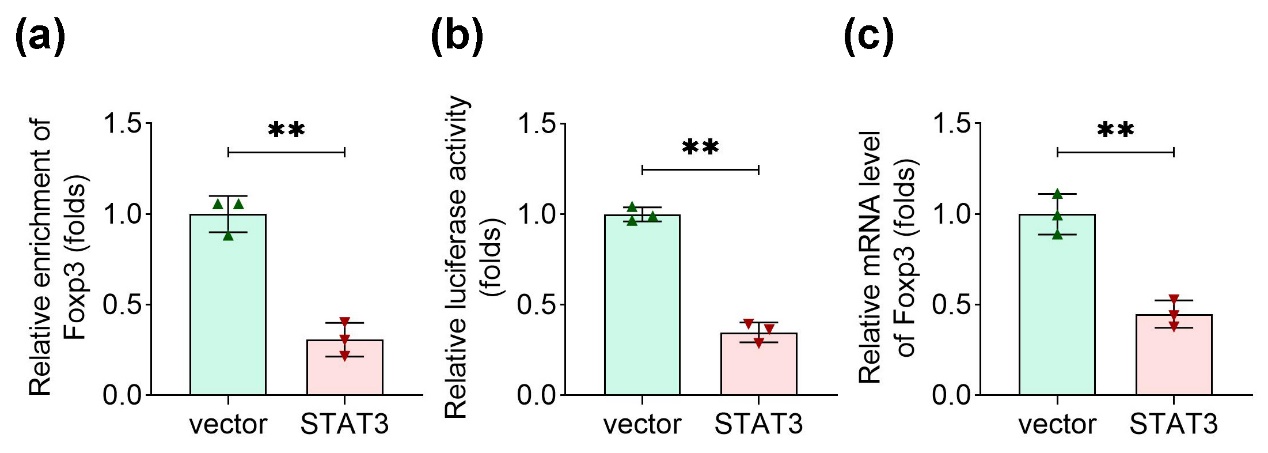


**Supplementary figure 2.** STAT3 inhibits the transcription of Foxp3. **(a)** The binding of STAT3 in the promoter region of Foxp3 was evaluated using ChIP. **(b)** The effect of STAT3 on transcription activity of Foxp3 was evaluated using luciferase reporter assay. **(c)** The regulation of STAT3 on Foxp3 mRNA expression was detected using qRT-PCR. n = 3 per group in each experiment. Each sample in each experiment was technically replicated three times. ** *P* < 0.01.

**Supplementary table 1.** Primer sequences used for quantitative PCR

| Name | Forward (5’-3’) | Reverse (5’-3’) |
| --- | --- | --- |
| ROR-γt | CTGAAAGCAGGAGCAATGGA | CGCTGAGGAAGTGGGAAA |
| Foxp3 | GTGGCCCGGATGTGAGAAG | GGAGCCCTTGTCGGATGATG |
| KAT2A | CTCTGCCTTAACTACTGGAAGC | GCCATCTGGTGTAATTGACCTTG |
| KAT3B | TTCCCCTAACCTCAATATGGGAG | GCCTGTGTCATTGGGCTTTTG |
| CPT1A | TCCAGTTGGCTTATCGTGGTG | TCCAGAGTCCGATTGATTTTTGC |
| SIRT5 | GCCATAGCCGAGTGTGAGAC | CAACTCCACAAGAGGTACATCG |
| SIRT7 | ACGCCAAATACTTGGTCGTCT | AGCACTAACGCTTCTCCCTTT |
| STAT3 | ACCAGCAGTATAGCCGCTTC | GCCACAATCCGGGCAATCT |
| GAPDH | CTGGGCTACACTGAGCACC | AAGTGGTCGTTGAGGGCAATG |
